# Serum Fluorescent Advanced Glycation End (F-AGE) products in gestational diabetes patients

**DOI:** 10.1590/2359-3997000000238

**Published:** 2017-01-27

**Authors:** João Paulo Lobo, Catiane Pompilio Brescansin, Izabella C. R. Santos-Weiss, Marciane Welter, Emanuel Maltempi de Souza, Fabiane Gomes de Moraes Rego, Geraldo Picheth, Dayane Alberton

**Affiliations:** 1 Departamento de Análise Clínica Universidade Federal do Paraná Curitiba PR Brasil Pós-Graduação em Ciências Farmacêuticas, Departamento de Análise Clínica, Universidade Federal do Paraná (UFPR), Curitiba, PR, Brasil; 2 Departamento de Bioquímica e Biologia Molecular UFPR Curitiba PR Brasil Departamento de Bioquímica e Biologia Molecular, UFPR, Curitiba, PR, Brasil

**Keywords:** Diabetes screening, gestational diabetes, fluorescent advanced glycation end products

## Abstract

**Objectives:**

Advanced glycation end products (AGEs) are involved in the pathogenesis and complications of diabetes mellitus (DM). Gestational DM (GDM) is characterized by increased glycemia and oxidative stress, which are factors associated with high serum AGE concentrations. The aim of this study was to evaluate the utility of a serum fluorescence AGE (F-AGE) method as a screening tool for gestational diabetes.

**Subjects and methods:**

Serum samples from 225 GDM patients and 217 healthy pregnant women (healthy controls) were diluted 50-fold in phosphate-buffered saline, and the AGEs were estimated by fluorometric analysis (λ_Ex_ 350 nm/ λ_Em_ 440 nm).

**Results:**

No significant (P > 0.05) differences in AGE concentrations, expressed in Arbitrary Units (UA/mL × 10^4^), were observed in the women with GDM or in the healthy controls. Furthermore, F-AGE concentrations did not change significantly during the pregnancy (12-32 weeks of gestation). Only the GDM group had a positive correlation (r = 0.421; P < 0.001) between F-AGEs and serum creatinine concentrations.

**Conclusion:**

It was not possible to distinguish women with gestational diabetes from the healthy controls on the basis of serum F-AGE concentrations.

## INTRODUCTION

Advanced glycation end products (AGEs) are generated by the non-enzymatic reaction of a sugar ketone or aldehyde group with the free amino groups of proteins, amino acids, lipids, and nucleic acids under conditions of hyperglycemia and oxidative stress ( 1 , 2 ). AGEs may cause tissue injury both directly, through phenomena such as trapping and cross-linking, and indirectly, by binding to specific receptors such as receptors for AGE (RAGE), which is expressed on the surface of numerous cell types, such as macrophages, monocytes, endothelial cells, neurons, and smooth muscle cells ( 3 , 4 ). The AGE-RAGE interaction can lead to oxidative stress, production of growth factors and cytokines, chronic inflammatory responses, and cellular and vascular dysfunction ( 5 , 6 ).

Elevated AGEs concentrations are associated with several diseases, including diabetes mellitus (DM) ( 5 , 7 , 8 ). DM is a pathology characterized by hyperglycemia, oxidative stress, inflammation, and consequently, the AGE-RAGE interaction is enhanced ( 1 , 5 ). While some studies have shown that AGE concentrations are higher in type 1 (T1D) and type 2 (T2D) diabetic patients than in healthy subjects, especially in diabetes with secondary complications ( 9 , 10 ), others have shown that AGE concentrations are also elevated in gestational DM (GDM) ( 11 , 12 ), and still other studies have demonstrated that AGEs concentrations were not significantly different between women with GDM and healthy pregnant women ( 13 , 14 ). However, a standard method to measure AGEs has not yet been established, making it difficult to compare results ( 15 ).

The absence of a universal method to measure AGEs is largely due to the characteristics of these compounds. AGEs constitute a large, complex, and heterogeneous group of molecules, and only some structures have been identified ( 1 , 16 ). ^ε^ *N* -carboxymethyl-lysine (CML), pentosidine, and methylglyoxal derivatives are examples of well-characterized AGEs ( 4 , 16 ). High performance liquid chromatography (HPLC), enzyme-linked immunosorbent assay (ELISA), immunohistochemistry and fluorescence spectroscopy have been used to measure the concentrations of the different types of AGEs ( 17 - 19 ).

Most AGEs have a characteristic fluorescence with an excitation maximum approximately at 370 nm and an emission maximum around 445 nm ( 20 ). Unlike other methods, fluorescence spectroscopy is rapid, cost effective and sample preparation is simple.

In this study, the fluorescence method was applied to measure the AGEs in the serum of pregnant women with GDM and healthy pregnant women in order to evaluate the screening capacity of this method and to examine the relationship AGEs concentration to other biochemistry parameters.

## MATERIALS AND METHODS

### Subjects

A total of 442 unrelated Euro-Brazilian pregnant women were examined. Healthy pregnant women were classified as controls (n = 217). Women with gestational diabetes (GDM, n = 225) were classified by the criteria of the Brazilian Diabetes Society – 2009 ( 21 ). Briefly, fasting plasma glucose ≥ 6.1 mmol/L and glycemia 2 h after 75 g oral glucose ≥ 7.8 mmol/L at 24^th^ – 28^th^ weeks of gestation. Patients with overt renal failure and cardiovascular disease were not included in the study.

The study was approved by the Federal University of Paraná’s Ethics Committee according to the Declaration of Helsinki, and all subjects gave written consent before measurements.

### Clinical and laboratory data

Clinical and anthropometric data were collected from patient files or from electronic patient registers. Fasting (8 h) blood was collected in Ethylenediaminetetraacetic Acid tripotassium salt tubes (K_3_EDTA, Vacutainer^®^, Becton Dickinson, New Jersey, USA) and Serum Separator tubes (Gel SST^®^ II Advance, Becton Dickinson, New Jersey, USA). The plasma and serum obtained were stored at -20 °C. The biochemical blood parameters were determined using an Architect Ci8200 system (Abbott Diagnostic Laboratory, Illinois, USA) with reagents, calibrators, and controls from the manufacturer ( Table 1 ).

**Table 1:** Anthropometric and laboratory characteristics of the study groups

Parameters	Control (n = 217)	GDM (n = 225)	P
Age (years)	29 (27–33)	32 (28–36)	< 0.001*
Weight (kg)	66.1 (58.5–73.8)	80.3 (70–93)	< 0.001*
Height (m)	1.61 ± 0.06	1.60 ± 0.07	0.003
BMI (kg/m^2^)	25.4 (22.5–28.3)	32.0 (27.7–36.4)	< 0.001*
Fasting glucose (mmol/L)	4.7 (4.4–4.9)	4.8 (4.6–5.4)	< 0.001*
Glucose 2h-75g (mmol/L)	4.8 (4.5-5.6)	9.0 (8.2–10.0)	< 0.001*
HbA1C (%)	-	5.6 (5.3–6.1)	-
Total Protein (g/L)	69 ± 7	63 ± 5	< 0.001
Albumin (g/L)	43 (38–46)	34 (32–36)	< 0.001*
Creatinine (µmol/L)	70.7 (61.9–79.6)	61.9 (53.0–70.7)	< 0.001*
AGE (AU/mL × 10^4^)	2.50 ± 0.86	2.42 ± 0.72	0.262
AGE (AU/g × 10^5^)	3.65 ± 1.15	3.84 ± 1.36	0.114

Values are presented as mean ± SD, median (interquartile range); - no information available.

Control, healthy pregnant women; GDM: gestational diabetes mellitus. P values, Student t-test (two-sided) or * Mann–Whitney U test.

### Fluorescent AGE assay

Measurement of fluorescent AGEs (F-AGEs) concentrations was based on the spectrofluorimetric detection ( 22 ). Serum was diluted 50-fold with phosphate-buffered saline (KH_2_PO_4_ 1.06 mmol/L, NaCl 155.10 mmol/L and Na_2_HPO_4_.7H_2_O 2.97 mmol/L, pH 7.4) and homogenized with vortex mixer for 10 seconds. The diluted serum (300 µL) was transferred into black 96-well plates. The excitation and emission wavelengths were 350 nm and 440 nm, respectively (Spectrofluorimeter Infinitive M200, TECAN, Mannedorf, Switzerland). PBS solution was used as blank. The fluorescence intensity was expressed in arbitrary units per milliliter of serum (AU/mL) and in AU/g of total protein. The total protein was measured by the biuret method (Architect Ci8200 system, Abbott Diagnostic Laboratory, Illinois, USA). The analytical coefficient of variation (CVa) was determined intra-assay as 5.1% (n = 15) and inter-assay as CVa = 7.9% (n = 22).

### Statistical analysis

The Kolmogorov-Smirnov test was applied to test the data for a normal distribution. Variables with a normal distribution were reported as mean ± SD and those with non-normal distribution as median (interquartile range, 25-75%). Comparisons between groups with continuous variables were tested with Student’s *t* test (independent) or the Mann–Whitney U test, as appropriate. ANOVA was used to compare more than two groups with normal distributions. The correlation analyses were carried out with Pearson’s correlation test. A P-value < 0.05 was accepted as the threshold for defining statistical significance. Statistical evaluation was performed with the Statistica software for Windows, version 8.0 (StatSoft Inc., Tulsa, OK, USA). ROC (Receiver Operating Characteristics) curves, cut-off points and the area under the curve (AUC) were calculated using MedCalc ver 12.2.1.0 (Mariakerke, Belgium).

## RESULTS

The clinical and laboratory characteristics of the healthy women and women with GDM pregnant patients are shown in Table 1 . The low fasting glucose and HbA1C concentrations suggested that the GDM patients had good glycemic control. Additionally, the low creatinine concentrations (< 106 µmol/L) indicate an absence of kidney damage.

**Figure 1:**
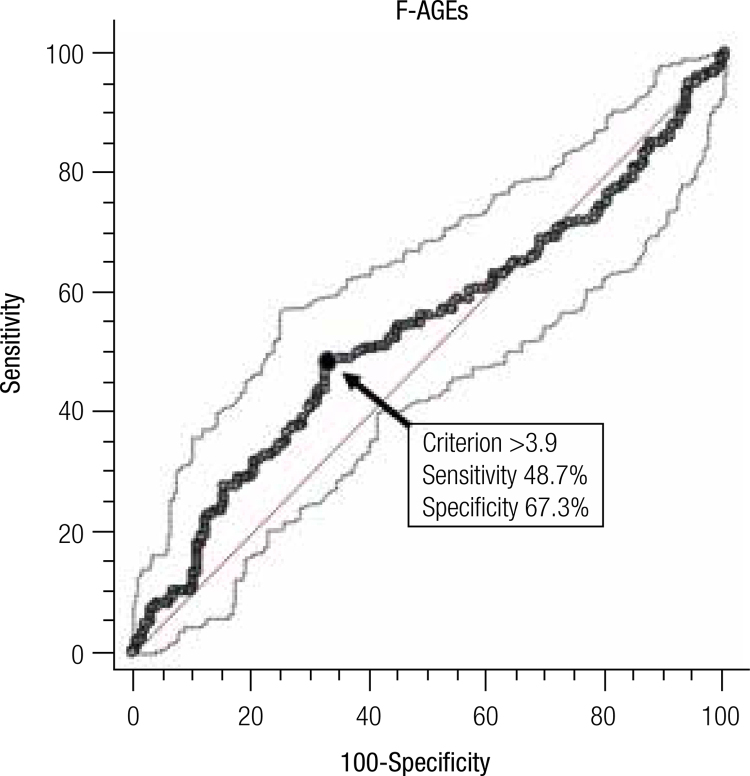
Receiver operating characteristic (ROC) curve for F-AGEs values in pregnant women with and without gestational diabetes. The AUC is 0.537 ± 0.036 (P = 0.188). The black circle (arrow) indicates the cut-off point (> 3.9 AU/g of protein) with sensitivity and specificity values of 48.7% and 67.3%, respectively. The dotted lines delimit the 95% confidence interval and the straight line is the line of equality.

The F-AGEs concentrations expressed as arbitrary units per unit volume (AU/mL) and per mass of protein (AU/g) were not statistically significant ( *P* > 0.05) between the two groups ( Table 1 ). ROC curve analysis ( Figure 1 ), with the area under the curve (AUC) of 0.537 ( *P* = 0.188), showed that the fluorescence assay did not have sufficient specificity (67.3%) and sensibility (48.7%) to classify the groups.

**Figure 2:**
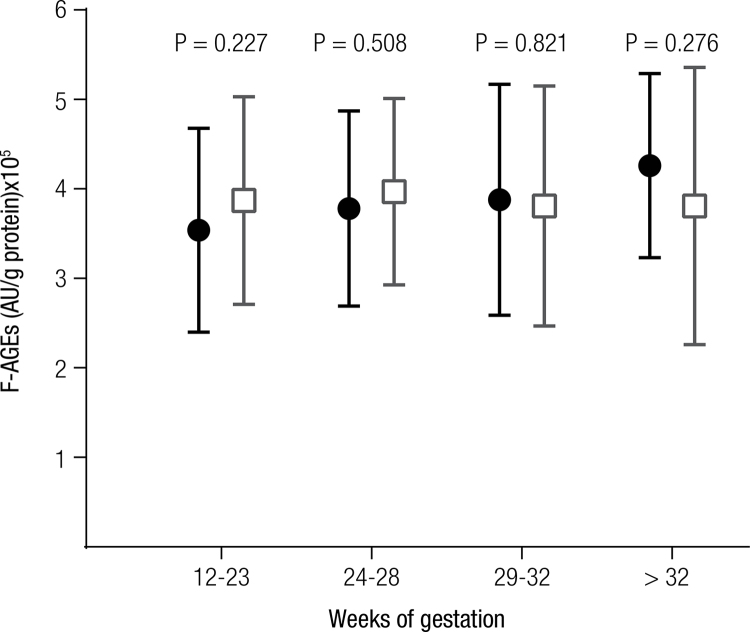
F-AGEs expressed in AU/g of protein were compared in four gestational periods. The results for healthy pregnant woman are shown as black circles and those for the GDM patients are represented by grey squares. The vertical bars represent 1-standard deviation. The P-values (Student’s t test) compared the F-AGE concentrations in the same gestational period. Variance analysis (ANOVA) did not show a significant difference for controls (P = 0.076) or GDM (P = 0.928).

**Figure 3:**
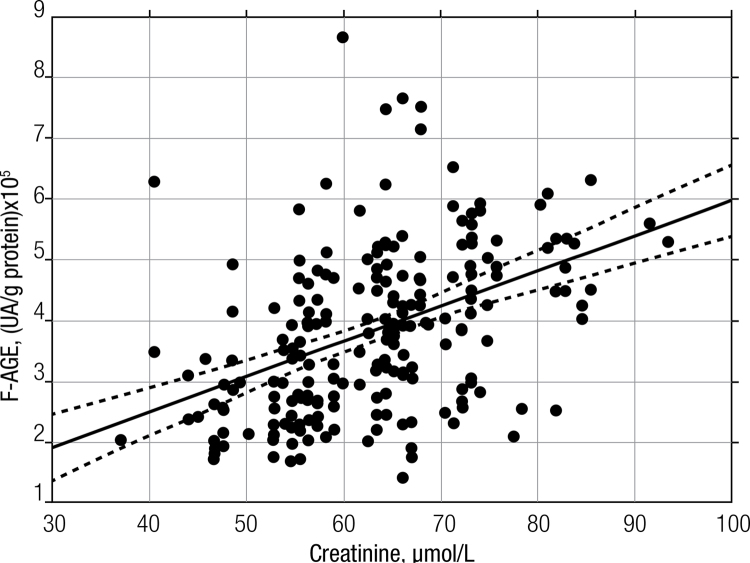
Linear correlation between serum creatinine and F-AGEs for the GDM group. Pearson’s Correlation (r = 0.421; P < 0.001) between F-AGEs and serum creatinine. The regression (solid) and 95% confidence interval (dotted) lines are shown.

The F-AGEs were also not significantly different in four gestational periods ( Figure 2 ). There was a significant positive correlation (r = 0.421; *P* < 0.001) between F-AGE concentration (UA/g protein) and serum creatinine concentrations in the GDM group ( Figure 3 ). Healthy pregnant women showed no correlation between F-AGE concentration and creatinine concentrations (r = 0.124; *P* = 0.049).

## DISCUSSION

Serum AGEs can be detected by many analytical methods, such as ELISA, radioimmunoassay, radioreceptor assay, fluorescence spectroscopy, and HPLC ( 18 , 20 , 23 , 24 ). Fluorescence spectroscopy is an easy and rapid method ( 22 ). Different studies have shown that the fluorescence assay (detecting F-AGEs) can be used to distinguish type 1 (T1D) and type 2 (T2D) diabetic patients from healthy subjects ( 19 , 22 ). The high oxidative stress conditions associated with diabetes likely play a more important role in AGE formation, in particular in type 2 diabetes, than the hyperglycemic state ( 22 ).

We postulated that GDM-induced mild hyperglycemia combined with oxidative stress could promote a significant increase in AGE concentrations when compared to that observed in healthy pregnant women. Therefore, we decided to evaluate the utility of a simple, fast, and inexpensive fluorimetric method to screen for GDM, where 96 samples could be processed in a short interval of time and with acceptable analytical performance (CVa < 8%; inter-assay). Our results showed that the proposed method could not be used to distinguish between the healthy patients and the GDM patients ( Table 1 ). The ROC curve analysis ( Figure 1 ) confirms that fluorescent AGEs were not able to efficiently discriminate the studied groups by the low sensibility and specificity observed.

These results are also consistent with a previous study, which showed that the skin autofluorescence AGE, measured using the AGE-Reader (DiagnOptics Technologies BV, Groningen, The Netherlands), also failed to distinguish GDM patients from healthy pregnant women ( 13 ). The authors justified this result due to mild severity and short duration of hyperglycemia in GDM at diagnosis. In our study, the good glycemic control observed in the GDM group (HbA1c 5.6%) likely explains the inability of fluorescence spectroscopy method to distinguish the GDM group from the healthy pregnant women. Therefore, we hypothesize that the presence of the mild hyperglycemia and oxidative stress in our GDM patients did not generate serum F-AGEs concentration enough to discriminate the groups studied. Buongiorno and cols *.* ( 11 ) differentiated GDM patients without adequate glycemic control from the control group by quantifying the AGE concentrations using the ELISA method, but could not differentiate women who previously had DM pregnancies and good glycemic control from healthy pregnant women.

In addition, in our population studied, no difference in F-AGEs was observed in the four major periods of gestation between the healthy women and women with gestational diabetes ( Figure 2 ). On the other hand, AGE concentrations measured by the fluorescence method in the serum of Chinese GDM pregnant women in mid-gestational and later gestational periods were also similar, but higher when compared to those of healthy pregnant women in the same gestational periods ( 25 ).

Of the correlations tested between F-AGE concentrations and biochemical blood parameters, the F-AGE was positively correlated with serum creatinine concentrations only in the GDM group ( Figure 3 ). Similar results were described for T1D and T2D patients (r = 0.84; *P* < 0.001) ( 26 , 27 ). In contrast, a significant positive correlation between low molecular weight serum F-AGEs and serum creatinine was shown in individuals with only minimal renal disturbance or with normal creatinine concentrations ( 28 ). Pentosidine (free form), an F-AGE, and possibly other AGEs are filtered through the glomeruli and reabsorbed in the proximal tubules ( 29 ). Therefore, decreased glomerular filtration rate and tubule cell damage could also be involved in AGE accumulation, as suggested by Wagner and cols. ( 27 ), who showed that patients with impaired renal function presented with increased serum CML and F-AGE concentrations and decreased creatinine clearance. In the present study, it is not clear why GDM pregnant women with normal serum creatinine concentrations presented with increased AGE concentrations, and more studies are necessary to determine the extent to which these findings are repeated elsewhere.

In summary, serum F-AGEs concentrations measured by fluorescence spectroscopy were not able to distinguish women with gestational diabetes from the healthy pregnant controls in our population.
